# Symmetric volume-reduction plasty of the enlarged left atrium: 15-years clinical experience

**DOI:** 10.1186/1749-8090-10-S1-A195

**Published:** 2015-12-16

**Authors:** Sergey L Dzemeshkevich, Vladimir V Raskin, Andrey S Dzemeshkevich, Yulia V Frolova, Sergey V Korolev, Maria S Malikova, Alexey N Lugovoy

**Affiliations:** 1 Department of Cardiac Surgery, Petrovsky National Research Centre of Surgery, Moscow, 119991, Russia

## Background/Introduction

Atriomegaly as a complication of mitral valve disease leads to atrial blood transport dysfunction, atrial fibrillation, respiratory insufficiency and heart failure.

## Aims/Objectives

To analyze the results of treatment of all concomitant cardiac pathologies including atriomegaly during original surgical correction of mitral valve disease.

## Method

From December 1998 to December 2013 we performed symmetric volume-reduction plasty of the left atrium (LA) in 104 patients with mitral valve disease using a technique we developed: we began the superior suture on the top of LA, above the line between the superior pulmonary veins; the right and left sutures were begun more laterally than the ostium of the inferior pulmonary veins, while on the left side we took into the suture the inner part of the left auriculum. All three sutures were tied up in the center of the posterior LA. Most of patients (87 cases) had a chronic form of atrial fibrillation and were in NYHA class IV. Mean age was 53,4+ 11 years. Procedures consisted of left atrium plasty (104 cases), mitral valve surgery (104 cases), tricuspid annuloplasty (47 cases), right atrium plasty (29 cases), aortic valve replacement (20 cases), left atrium thrombectomy (7 cases), coronary artery bypass grafting (5 cases), artificial chorde implantation (5 cases).

## Results

There were 2 in-hospital deaths (1,9%). The size of left atrium after surgery decreased from 8,3+ 2,1x 6,7+ 1,4 cm (range 5 to 14,5 cm) to 5,0+1,3x4,7+ 1,1 cm. The cardiothoracic ratio decreased from 0,62+ 0,04 to 0,53+ 0,02. The tracheal bifurcation angle decreased from 94,3+ 5,3° to 76,0+ 6,3°. The follow-up data from 12 months to 15 years were available for 89 pts, and 54 pts (60.7%) among them reported the sinus rhythm.

## Discussion/Conclusion

Clinical, functional and anatomic results support the use of this original surgical technique of the symmetric atrial plasty for treatment of enlarged left atrium in patients undergoing mitral valve surgery. These data support the critical mass hypothesis of atrium fibrillation.

